# The Book World of Medicine and Science

**Published:** 1907-09-14

**Authors:** 


### The Book World of Medicine and Science.

### STRAY NOTES.

**The London Matriculation.**

At the present time, when so much is being written in the medical press about the virtues and demerits of the entrance examination of the London University, a good hand-book to the examination will prove of real benefit to the intending "London" medical candidate. The latest edition of the "University Tutorial Series Matriculation Directory" is in every way an efficient guide book, and may be confidently commended. It is published at the low price of one shilling and contains a vast amount of useful and reliable information.

**A Disappointment.**

The previous volumes of the "Medical Epitome Series" issued by Messrs. Hodder and Stoughton, of Warwick Square, E.C., have been so excellent that the latest addition to the series, "Diseases of the Nose and Throat," by Dr. J. Bruce Ferguson (4s. net), comes as a disappointment. Perhaps, judging from the previous volumes, we expected too much, but it is unfortunate that the editor of what promised to be an excellent series of helpful little manuals should have spoilt the good reputation of the whole by including in it a work of such indifferent merits as that just issued. Let us hasten to say that the fault is more that of the publisher—or rather the printers' reader—than of the author. Dr. Ferguson's teaching, though necessarily brief, is usually sound. Here and there he "overloads" by unnecessary detail, and we must protest against his prescriptions, in many of which trade names are given. At times, too, he is not clear. In his description of frontal sinusitis, for instance, no mention is made of the frequent association of this condition with nasal polypi. But these faults are slight compared with the slovenly way in which the whole booklet has been subedited. Printers' errors are irritably frequent, and at times completely destructive to the reader's patience. In a future edition, too, we venture to suggest that the term "Nose-bleed" be dropped. It is doubtless short enough, but it jars.

**The Cinematograph.**

In "The Cinematograph in Science, Education, and Matters of State" Mr. Charles Urban, F.Z.S., gives a condensed summary of the uses to which this invention may be put. "The entertainer," he remarks—and everyone will recognise how much truth underlies his statement—"has hitherto monopolised the cinematograph for exhibition purposes, but movement in more serious directions has become imperative." He proceeds to show how valuable an addition to ordinary educational methods the instrument may be. "Major surgical operations . . . are caught in every phase by the camera and held for reference at any time." At the present moment in Paris there is a cinematograph exhibition of important surgical operations which may be seen by anyone willing to pay the 50 centimes entrance fee (children excluded); and, so far as we have been able to see, the productions, though perhaps not judiciously selected, are as a rule faithful. Whether they serve any useful purpose is, however, to be questioned. But the fact that the crowd gathered round the exhibition on the Champs de Mars is attracted solely by motives of morbid curiosity, does not argue against Mr. Urban's main contention that the cinematograph has educational possibilities which we have hitherto inadequately recognised. His little pamphlet is worth studying. It may be obtained gratis on application to the Urban Trading Co., Limited, 48 Rupert Street, W.

**Chemical Physiology.**

Dr. Halliburton's "Essentials of Chemical Physiology" is so well known that a new edition of this familiar work needs little in the way of introduction. The sixth edition (Longmans, Green, London, 4s. net) is in every way a useful text-book, eminently practical and up-to-date. Following the more advanced method, Professor Halliburton has dropped the word "proteid" and substituted the more just title "protein." He adopts Fischer's classification, and the chapter on the properties of this large class of substances is one of the best in the book. The advanced work, to which a special section is devoted, is fully dealt with. Methods of blood examination are lucidly described. There are a few points included which are of less clinical than purely physiological importance, but, after all, the book is intended for the working student, not for the practising physician. The latter, however, may find much information in it, and it is a useful little volume to have at hand.

**A Chapter on the History of Anæsthetics.**

The fourth edition of Dr. Dudley W. Buxton's "Anæsthetics: Their Uses and Administration"—one of Mr. Lewis's excellent "Practical Series," published at 7s. 6d. net—contains several fresh articles which make the information up-to-date in every respect. The new regulators are fully described, and mention is made of the Dubois and Levi apparatus. Special attention has been paid to the chapters dealing with local anæsthesia, and the newer methods, such as the scopolamine and stovaine injections, are referred to. The book contains a very excellent, though necessarily brief, *résumé* of the history of anæsthetics, which is accurate and informative. It is a feature which might advantageously be copied by other text-books. Medical history is too often neglected by the student, and we venture to think that such articles as that with which Dr. Buxton opens his book serve a very useful purpose in impressing certain facts upon the reader's mind. This text-book is to be heartily commended, for it is one of the best of its kind that we have met.

**A Consultant's Directory.**

The Scientific Press, Ltd., 28 and 29 Southampton Street, has been well advised in issuing the modest "Medical Homes for Private Patients, 1907: A Classified Directory with Lists of Medical Consultants and Specialists." The directory, which gives very full information regarding all the nursing homes and consumption sanatoria, contains a list of all the consultants in the United Kingdom, with their addresses. In a future edition the telephone numbers of specialists might with advantage be added. The price of the directory is sixpence net.

**Poisons and Poisoning.**

In 1881 Mr. H. K. Lewis, 136 Gower Street, W.C., published the first edition of Dr. William Murrell's little work, "What to do in cases of Poisoning," and the fact that the tenth edition has just been issued speaks well for the popularity of the tiny volume. The new edition is a masterpiece of condensation and brevity. A great deal more, for instance, might have been written on the subject of carbolic acid poisoning (for which, by the way, a new antidote has been claimed in tincture of iodine), but the essence of the whole literature on the subject is admirably given by Dr. Murrell. A complete, accurate, and thoroughly reliable guide such as the book is cannot fail to command respect and a cordial welcome, and to those who wish for a handy reference work we can recommend Murrell's Poisons.

